# Food responsiveness, addiction, and hyperphagia in Prader-Willi syndrome: a cross-sectional study of 210 Chinese patients

**DOI:** 10.3389/fendo.2025.1665040

**Published:** 2025-10-23

**Authors:** Chen-Xi Hu, Fang-ling Xia, Yi-Fang Qin, Yun-Qi Chao, Yu-Lu Ruan, Jing-Wen Li, Guan-Ping Dong, Chao-Chun Zou

**Affiliations:** Department of Endocrinology, Children’s Hospital, Zhejiang University School of Medicine, National Clinical Research Center for Child Health, Hangzhou, Zhejiang, China

**Keywords:** Prader-Willi syndrome, hyperphagia, obesity, food addiction, food responsiveness, growth hormone treatment

## Abstract

**Objective:**

Prader–Willi syndrome (PWS) is the most common genetic syndromic obesity, characterized primarily by hyperphagia. It was described as excessive appetite, defective satiety, and obsession with food. However, the underlying mechanisms of hyperphagia in PWS remain obscure. This study aimed to determine the eating behavior patterns and food addiction tendencies in patients with PWS.

**Methods:**

210 patients with PWS from 26 provinces in China were enrolled in this study. The translated Children’s Eating Behavior Questionnaire and modified Yale Food Addiction Scale for Children 2.0 were adopted for evaluation.

**Results:**

This study revealed that (i) In patients with PWS, the eating behavior patterns are abnormal and the risk of food addiction is high. (ii) Patients with higher food responsiveness (FR) have a higher risk of food addiction; (iii) Scores of food responsiveness, enjoyment of food, satiety response, and food addiction have already changed even before the onset of overweight or obesity. (iv) Growth hormone (GH) therapy is an independent factor influencing weight, with continuous treatment being beneficial for weight maintenance and earlier treatment being more advantageous. (v) FR is another key factor affecting body weight. Unfortunately, GH therapy does not improve food responsiveness.

**Conclusions:**

This study indicates that GH treatment and FR are significant factors influencing hyperphagia and body weight in patients with PWS. Early involvement of psychotherapeutic interventions may help patients better manage hyperphagia-related behaviors and subsequent weight gain.

## 1 Introduction

Prader–Willi syndrome (PWS) (1) is the most common genetic syndromic obesity, a neurodevelopmental disorder resulting from errors in a complex genomic mechanism known as genomic imprinting. The main clinical symptoms and characteristics are poor suck and feeding difficulties caused by severe hypotonia during infancy, followed by hyperphagia, obesity, and obesity-related complications. In general, it is also accompanied by distinctive appearance, hypogonadism/hypogenitalism, motor and cognitive delays, and other endocrine abnormalities (2–4). This disorder is classified into three molecular genetic categories:(i) Paternal deletion of the 15q11-q13 region, occurring in 60–70% of cases. (ii) Maternal uniparental disomy 15, where both copies of chromosome 15 are inherited from the mother, is found in approximately 25–35% of cases. (iii) Defects in the imprinting center, which regulates the imprinted genes on chromosome 15 (5, 6), affect 1–3% of cases.

PWS can be divided into four nutritional phases (7, 8): Phase 0 is characterized by reduced fetal movements and growth restriction in the uterus; Phase 1a mainly features hypotonia with or without failure (birth to 9 months) and phase 1b features normal growth development(9~25 months); in Phase 2, the initial sign is weight gain without a significant change in appetite or caloric intake (median age of onset: 2.08 years), followed by increased interest in food and further weight gain (median age of onset: 4.5 years). Phase 3 is characterized by hyperphagia, which is often accompanied by food-seeking behavior and a lack of satiety (median age of onset: ~8 years). The main causes of death for patients with PWS are respiratory failure, cardiac failure, and hyperphagia-related accidents (9, 10). Multiple studies have shown that many diseases, such as obesity, cardiovascular mortality, and even chronic kidney disease, are related to dietary factors (11–13). PWS is a disease associated with eating disorders. Thus, the eating behavior of PWS patients deserves great attention. Moreover, most patients with PWS require constant diet restriction and supervision in a controlled environment, which brings a great burden on parents and caregivers. (14) Hence, appetite control is essential for patients with PWS and their caregivers.

Ghrelin is a gastrointestinal hormone that stimulates hunger. Several studies have shown that patients with PWS with hyperphagia, particularly older children and adults, have significantly elevated levels of ghrelin before and after meals (15), which led to the view that hyperghrelinemia could be a cause of hyperphagia. However, the application of antagonists of the ghrelin receptor in a murine model of PWS reveals its ineffectiveness in terms of appetite control. Furthermore, clinical trials of medications that decrease the level of active ghrelin in blood have shown no improvement in hyperphagia scores (16, 17). Until now, the underlying cause of hyperphagia remains obscure.

Eating disorders in patients with PWS can be described as excessive appetite, defective satiety, and obsession with food. Juliette Salles et al. suggest that hyperphagia in patients with PWS can be described as food addiction-like behaviors or food addiction (18). Food addiction is a complex condition characterized by intense cravings for food despite harmful consequences. Adams et al. (19) describe it as a cycle that initially manifests as vulnerability to excessive consumption of tasty foods, characterized by (i) increased impulsivity, (ii) reward sensitivity, and (iii) decreased inhibitory control. As a result of excessive food consumption, individuals with food addiction develop tolerance, cravings, and withdrawal, along with a range of social, emotional, and behavioral difficulties. Notably, these behaviors are also observed in patients with PWS, such as stealing food and eating garbage, and are included in the assessment of appetite in the PWS hyperphagia questionnaire (20). Through functional magnetic resonance imaging, multiple brain nuclei in individuals with PWS respond abnormally to peripheral signals, especially in response to high-calorie food. These regions include the hypothalamus, hippocampus, amygdala, and nucleus accumbens, which may indicate some defects and impairments in satiety and reward systems. (21). All of the above suggests the possibility of food addiction contributing to hyperphagia for patients with PWS.

Impaired growth rate, short stature, low energy expenditure, and abnormal body composition are common in Prader-Willi syndrome patients, related to growth hormone deficiency (22). Growth hormone therapy is the only medication approved by the FDA for the treatment of Prader-Willi Syndrome (2). Multiple studies demonstrated that children with PWS could have normal adult height, better body composition, and motor ability with treatment with GH, without adverse effect on metabolism and bone maturation (23, 24). More extensive studies indicate that earlier GH therapy also benefits the cognitive and linguistic development in patients with PWS (25). However, its influence on appetite is poorly understood.

Here, we use the Children’s Eating Behavior Questionnaire (CEBQ) and modified Yale Food Addiction Scale 2.0 (mYFAS-C 2.0) to evaluate the eating behavior pattern and risk of food addiction in patients with PWS, and evaluate the benefit of GH therapy on appetite. We hope to provide data and guidance for clinical intervention and new perspectives for scientific research.

## 2 Materials and methods

### 2.1 Participants

Electronic informed consent was obtained from every participant/guardian. Before accessing the questionnaire, all respondents were required to (a) know about the study purpose, voluntary participation, and withdrawal rights. (b) Actively check the agreement box and carry out the next step of research by choice.​ This protocol was approved by the ethics committee as a valid consent procedure. This study was reviewed and approved by the Ethics Committee of the Children’s Hospital of Zhejiang University, School of Medicine, which strictly adheres to the ethical principles of the Declaration of Helsinki (Approval number: 2024-IRB-0240). A total of 218 patients with genetically confirmed PWS from 26 provinces in China completed the questionnaires. Children with missing information or children whose caregivers refused to return visits were excluded from the research. Finally, 210 PWS patients, together with their caregivers, were enrolled in this study. The reference data of the healthy control group are detailed in (26).

### 2.2 Children’s Eating Behavior Questionnaire

The CEBQ (27) is a widely used parent-reported instrument designed to assess various dimensions of eating behavior and identify specific eating behaviors that can contribute to obesity in children. The Chinese version of the CEBQ was translated by Doctor Xue and verified in 1,500 primary school students aged 7–10 years; it has been shown to have good internal consistency, retest reliability, and reasonable structural validity. The detail was described in (28). With the original author’s consent, the Chinese version of the CEBQ is adopted.

The CEBQ comprises 35 questions divided into 8 subscales that assess different aspects of children’s eating behavior, including food responsiveness (FR), enjoyment of food (EF), satiety responsiveness (SR), slowness in eating (SE), emotional over-eating (EOE), emotional under-eating (EUE), desire to drink (DD), and food fussiness (FF). Each subscale consists of 3–6 items.

Scores were assessed via a Likert scale ranging from 1 to 5, as follows: [1] never, [2] rarely, [3] sometimes, [4] often, [5] always. The higher the score in each dimension is, the greater the underlying characteristic of a certain eating behavior. In this study, the reliability of the CEBQ was 0.709, and each subscale ranged from 0.664 to 0.897. For detailed factor analysis, see **Table 1**. The average score for each dimension was calculated for further analysis.

**Table 1:** The influence of gender, age, genetic subtype, weight, and GH therapy in the scores of CEBQ subscale and mYFAS-C 2.0.

Eating behaviors	Gender(H,P)	Age group(H,P)	Genetic subtype(H,P)	Weight categories(H,P)	GH therapy(H,P)	Cronbach’s α
CEBQ						0.709
FR	5245 (0.542)^##^	38.476 (<0.001***)	12.694 (0.002**)	24.359 (<0.001***)	10.434 (0.005**)	0.897
EF	5367 (0.736)^##^	33.707 (<0.001***)	9.292 (0.01*)	18.682 (<0.001***)	5.301 (0.071)	0.868
SR	5158 (0.418)^##^	23.504 (<0.001***)	8.205 (0.017*)	9.175 (0.01*)	3.788 (0.15)	0.764
SE	4116 (0.001)^##^	10.078 (0.018*)	0.66 (0.719)	10.865 (0.004**)	5.053 (0.08)	0.819
EOE	5440 (0.869)	18.721 (<0.001***)	12.508 (0.002**)	20.456 (<0.001***)	8.908 (0.012*)	0.836
EUE	5450 (0.886)	4.801 (0.187)	3.56 (0.169)	1.727 (0.422)	1.656 (0.437)	0.588
DD	5484 (0.948)	6.919 (0.075)	3.611 (0.164)	5.441 (0.066)	8.361 (0.015*)	0.852
FF	0.423 (0.516)^#^	20.432 (<0.001***)	9.934 (0.007**)	4.126 (0.02*)^#^	1.724 (0.422)	0.664
mYFAS2.0	5461 (0.907)^##^	30.199 (<0.001***)	7.532 (0.023*)	11.623 (<0.001***)^#^	6.788 (0.002**)^#^	0.927

^#^One-way ANOVA test. ^##^Mann-Whitney U test. The rest are the Kruskal-Wallis test. *P < 0.05, **P < 0.01, ***P < 0.001.

FR, food responsiveness; EF, enjoyment of food; SR, satiety responsiveness; SE, slowness in eating; EOE, emotional over-eating; EUE, emotional under-eating; DD, desire to drink; FF, food fussiness.

### 2.3 Modified Yale food addiction scale for children 2.0

The mYFAS-C (29) was used to measure addictive-like eating behavior. It is a self-assessment scale consisting of 11 questions measured on a Likert-type scale (0 = “never” to 4 = “always”). The severity of food addiction can be assessed based on the total score of the responses. The higher the total score, the greater the severity of food addiction. Dr. Wang (30) translated it into Chinese and confirmed its good reliability and validity among 426 children and adolescents in the Chinese population. The mYFAS-C 2.0 score showed acceptable internal consistency (Cronbach’s α = 0.927) in this study. The content validity index (CVI) of the scale was 0.97 (using the averaging method). For detailed factor analysis, see **Table 1**.

### 2.4 Body mass index z score

Children and adolescents’ weight and height were measured via standard methods by nurses. The BMI-Z score was calculated using the WHO AnthroPlus software. The standards of the World Health Organization (WHO) were used to calculate each child/adolescent by age and gender. (31) 1<BMI-Z ≤ 2 indicates overweight while BMI-Z>2 indicates obesity.

### 2.5 Data analysis

Statistical analysis was performed via SPSS 27.0 software. Categorical data were expressed as percentages (such as the percentage of gene subtype), while continuous variables were first subjected to a normality test. Data that followed a Gaussian distribution were presented as mean ± standard deviation (x ± s). Data that followed skewed distribution were presented as median and quartile.

To verify the underlying structure of the Chinese version of the questionnaire and determine whether it was similar to the original CEBQ (32), Principal Components Analysis (PCA) with direct oblimin rotation was performed on all 35 CEBQ items. The factor load was set to 0.4 (27) as the threshold for factor analysis. Cronbach’s α was used to evaluate the internal consistency of the questionnaire.

Eight subscales of the CEBQ and mYFAS-C 2.0 were compared by age group via the Kruskal-Wallis test. Welch’s test was used to analyze continuous and normally distributed data. Pearson’s and Spearman’s correlations were used to analyze the correlations among the variables. Based on the data distribution characteristics, One-way ANOVA, Mann-Whitney U test, and Kruskal-Wallis test were used to compare the values of various CEBQ subscales, the mYFAS-C 2.0 scores across different groups. Multiple linear regression analysis was used to examine the relationships between BMI-Z scores and various CEBQ items. *P* values <0.05 were considered statistically significant.

## 3 Results

### 3.1 Demographics and anthropometrics

A total of 105 (50%) men and 105 (50%) women were enrolled in this study;155 (73.8%) had paternal deletion, 33 (15.8%) had non-deletion subtype including maternal uniparental disomy and imprinting defect, and 22 with unknown subtype, mainly detected by methylation-specific PCR(MS-PCR). The mean age was 7.14 ± 4.05 years. According to nutritional phases in Prader-Willi syndrome (PWS), patients were divided into 4 groups: < 2 years (11.9%), 2–5 years (29.5%), 5–8 years (26.7%), and > 8 years (31.9%). Among the participants, 76 (36.2%) were normal weight, 26 (12.4%) were overweight, and 108 (51.4%) were obese. Other descriptive statistics and comparisons are listed in **Table 2**.

**Table 2:** Demographic characteristics of participants (N = 210).

Characters	N(%)
Gender	
Female	105 (50%)
Male	105 (50%)
Age group	
<2 years	25 (11.9%)
2-5 years	62 (29.5%)
5-8 years	56 (26.7%)
8 years-	67 (31.9%)
Genetic subtype	
Deletion	155 (73.8%)
Non-deletion	33 (15.8%)
Not clear	22 (10.5%)
Weight categories	
Normal weight	76 (36.2%)
Overweight	26 (12.4)
obesity	108 (51.4%)
	**Mean (SD), median**
Age (years)	7.14 (4.05), 6.5
BMI-Z	2.73 (3.61),2.17

### 3.2 The eating behavior patterns are abnormal in patients with PWS

**Figure 1:**
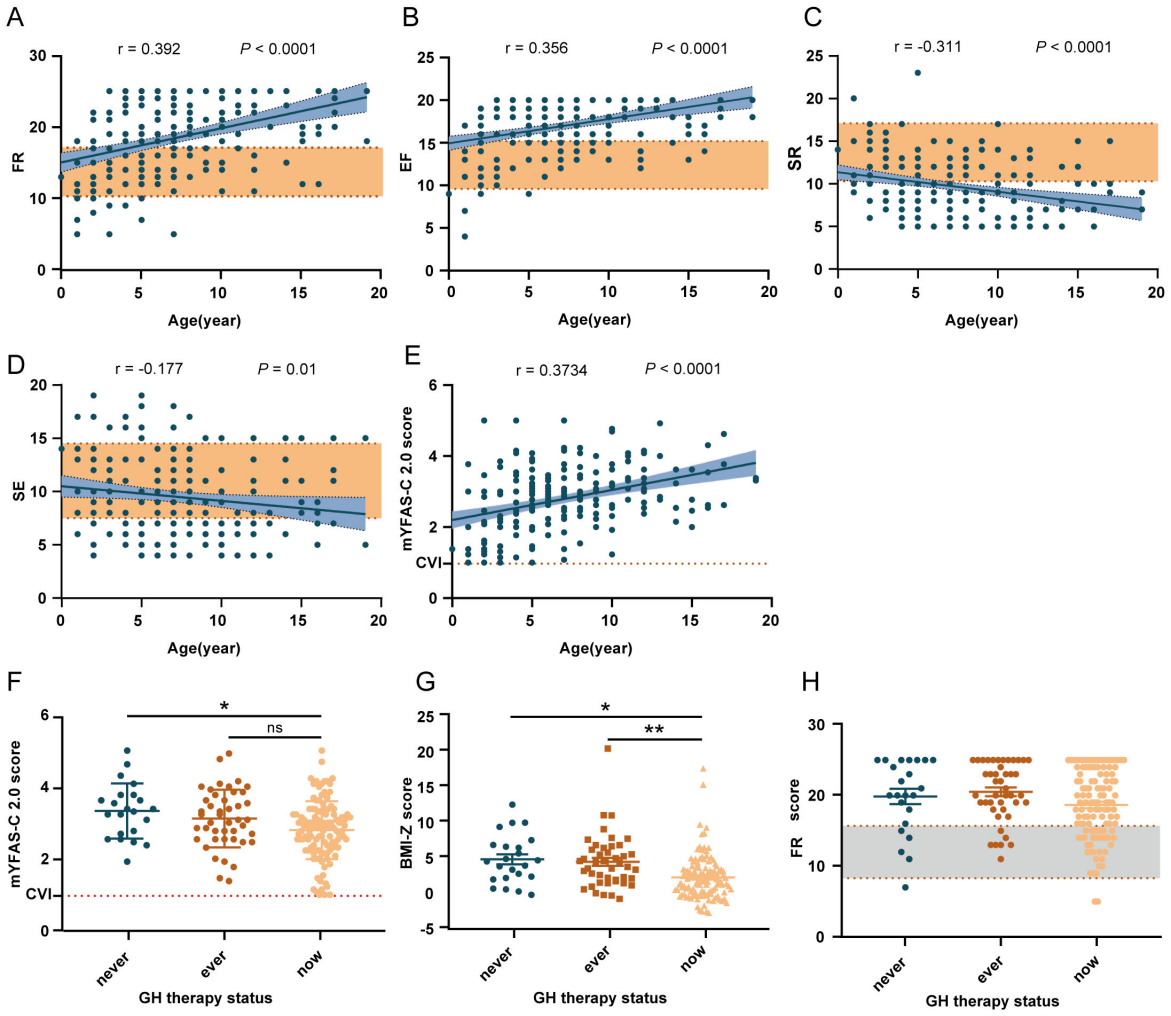
Factors related to the scores of FR, EF, SR, SE, and the mYFAS-C 2.0. **(A–E)** Trends in the scores of FR, EF, SR, SE, and the mYFAS-C 2.0 with age in PWS patients. The yellow shading represents healthy controls’ data. **(F–H)** The influence of GH therapy on mYFAS-C 2.0 score, FR score, and BMI-Z. *P < 0.05, **P < 0.01, ***P < 0.001. FR, food responsiveness; EF, enjoyment of food; SR, satiety responsiveness; SE, slowness in eating.

Through the analysis, we found that FR, EF, and mYFAS-C 2.0 scores were higher, while SR was lower in patients with PWS than healthy controls. Further age related linear regression analysis showed (**Figures 1A–D**) that SR(r = 0.2793, *P* < 0.0001) and SE (r = 0.1492, *P* = 0.03) decreased with age while FR (r = 0.3707, *P* < 0.0001), EF (r = 0.3579, *P* < 0.0001) and mYFAS-C 2.0 scores (r = 0.3734, *P* < 0.0001) increased with age in patients with PWS. The results indicate that patients with PWS might have a higher response to environmental food cues, a higher risk of food addiction, and lower control over food intake to meet energy needs compared to controls.

Then, we investigated whether gender, age, genetic subtype, body weight, or growth hormone(GH) therapy plays a role in each Children’s Eating Behavior Questionnaire (CEBQ) subscale scores and Modified Yale food addiction scale for children 2.0 (mYFAS-C 2.0) scores. Among these factors, Age and body weight might be correlated with CEBQ subscale scores and mYFAS-C 2.0 scores (**Table 1**).

### 3.3 Patients with higher FR have a higher risk of food addiction

According to clinical characteristics, food addiction might contribute to the obsession with food observed in PWS, such as hoarding, compulsive food searching, and other related behaviors. As shown in **Figure 1E**, the mYFAS-C 2.0 score was significantly higher in patients with PWS than in healthy controls, indicating a higher risk of food addiction in patients with PWS.

The correlations between scales suggest that food addiction is significantly positively correlated with FR, EF, and EOE, and significantly negatively correlated with SR, SE, and FF (**Supplementary Table S1**). To elucidate the factors correlated with mYFAS-C 2.0 scores, we constructed stepwise linear regression analysis with mYFAS-C 2.0 scores as the outcome variable and other factors, such as CEBQ subscale scores and demographic characteristics, as independent variables. Through hierarchical linear regression analyses for the mYFAS-C 2.0 score (**Table 3**), we found that the mYFAS-C 2.0 score was primarily and significantly correlated with FR, which means patients with higher FR have a higher risk of food addiction. Notably, GH therapy did not significantly improve mYFAS-C 2.0 scores (**Figure 1F**).

**Table 3:** Hierarchical linear regression step-by-step analyses all factors on mYFAS-C 2.0.

	Standardized β	95% CI for standardized β		P-value
		Lower bound	Upper bound	
FR	0.457	0.048	0.107	<0.001***
EOE	0.259	0.03	0.083	<0.001***
EF	0.199	0.015	0.109	0.009**
FF	0.17	0.011	0.056	0.004**
age	0.124	0.005	0.05	0.015*

*P < 0.05, **P < 0.01, ***P < 0.001.

FR, food responsiveness; EF, enjoyment of food; EOE, emotional over-eating; FF, food fussiness.

### 3.4 Early alterations of FR, EF, and food addiction may contribute to hyperphagia in patients with PWS

**Figure 2:**
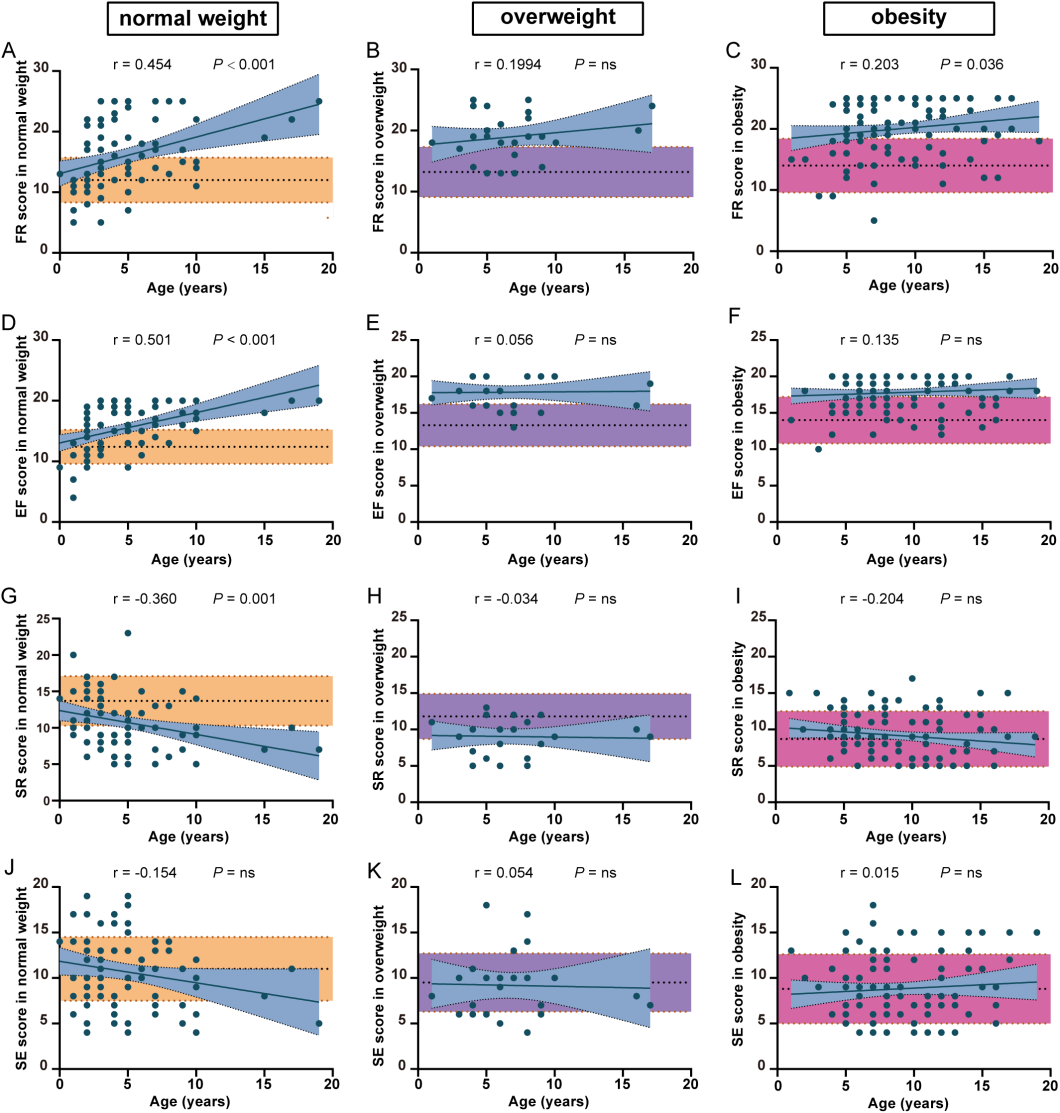
The comparison of eating behavior between PWS patients and healthy controls among different weight categories. The difference in FR **(A–C)**, EF **(D–F)**, SR **(G–I)** and SE **(J–L)** scores between PWS patients and healthy controls among different weight categories. The yellow, purple and red shades represent healthy controls with normal weight, overweight and obesity. FR, food responsiveness; EF, enjoyment of food; SR, satiety responsiveness; SE, slowness in eating.

Due to the analysis shown in **Supplementary Table S2** and the influence of body weight on CEBQ subscale scores (26), we then explored the differences in some CEBQ subscales between patients with PWS and healthy controls among different weight categories. What is surprising to us is that in the obesity groups, FR and EF scores in patients with PWS were still higher than in the healthy controls (**Figures 2C, F**), while SR and SE scores in patients with PWS were similar in the healthy controls. (**Figures 2I, L**) These results indicate that the abnormalities of FR and EF are specific to patients with PWS. More, FR and EF scores were significantly higher than controls (**Figures 2A, B**), and SR was lower than controls in the normal and overweight groups (**Figures 2D, E**), meaning the eating behavior pattern alters much earlier in patients with PWS. Together, these phenomena suggest that elevated FR and EF might contribute to hyperphagia in patients with PWS. Similarly, mYFAS-C 2.0 scores have already exhibited well above the standard threshold in the normal weight group of patients with PWS, indicating that food addiction might be involved in the progression of hyperphagia in PWS (**Supplementary Figure S1A**).

### 3.5 GH therapy and food responsiveness are key factors in weight management for patients with PWS

Obesity and obesity-related disorders present significant challenges to the lifespan of patients with PWS. We wonder whether the alterations in eating behavior are correlated with body weight. Hence, we aimed to identify factors influencing body weight in patients with PWS.

Notably, we found that scores before 2 years were significantly different from those of other age groups. (**Supplementary Table S3**, **Supplementary Figures S1B–F**) Considering the characteristics of the nutritional phases in PWS, subsequent analyses primarily focused on data collected after 2 years of age. Through correlation analysis, “food approach” subscales (FR, EF, and EOE) are negatively correlated with “food avoidant” subscales, which is consistent with previous studies. (14, 33, 34) (**Supplementary Table S1**) BMI-Z was strongly positively correlated with age, mYFAS-C 2.0 score, FR, EF, and EOE, and negatively correlated with GH therapy, SR, SE, and FF.

Using the Kruskal-Wallis test, we found that weight categories were related to age, genetic type, and GH therapy status. The prevalence of obesity was lower in the ‘on GH therapy’ group compared to the other two groups (51.4% in the ‘on GH therapy’ group versus 71.1% in the ‘ever on GH therapy’ group and 76% in the ‘never on GH therapy’ group) (**Supplementary Table S4**) (**Figure 1G**). Subsequently, we conducted a separate analysis of the contribution of GH therapy to BMI-Z (**Table 4**). We found that an earlier initiation age of GH therapy and adherence to the treatment had a positive effect on weight management.

**Table 4:** Hierarchical linear regression analyses of initial age and duration of GH therapy.

	Standardized β	95% CI for standardized β		P-value
		Lower bound	Upper bound	
Initial age	0.197	0.034	0.368	0.018*
duration	0.066	-0.138	0.327	0.424

*P < 0.05.

To elucidate the primary factors influencing BMI-Z while controlling for confounding variables, we constructed a stepwise linear regression analysis to estimate the partial regression coefficients (**Table 5**). We used age, gender, GH treatment, various CEBQ subscale scores, or mYFAS-C 2.0 scores as independent variables, with BMI-Z as the dependent variable. This analysis revealed that BMI-Z is predominantly associated with GH therapy and FR (*P* < 0.001 and *P* = 0.001, respectively) and was not associated with age, genetic type, EF, or mYFAS-C 2.0 scores. Unfortunately, GH therapy does not reduce food responsiveness (**Figure 1H**).

**Table 5:** Hierarchical linear regression step-by-step analyses of all factors on BMI-Z score.

	Standardized β	95% CI for standardized β		P-value
		Lower bound	Upper bound	
GH therapy	-0.319	-1.85	-0.774	<0.001***
FR	0.238	0.059	0.221	0.001**
SE	-0.179	-0.248	-0.034	0.01*

*P < 0.05, **P < 0.01, ***P < 0.001.

FR, food responsiveness; SE, slowness in eating.

## 4 Discussion

Patients with PWS are characterized by hyperphagia after 8 years (4, 35), which is commonly described as an insatiable appetite and an inability to feel full, possibly linked to hyperghrelinemia and a reduction in oxytocin-secreting neurons. (22) In 2021, Juliette Salles et al. proposed that food addiction might play a role in hyperphagia in PWS. (18) Several studies have reported abnormalities in the reward/limbic regions, most of which were identified using brain imaging techniques. (36–40) However, evidence for food addiction in patients with PWS remains limited. In our research, we provided evidence that patients with PWS have an abnormal eating behavior pattern and a higher tendency toward food addiction for the first time. Patients with higher FR tend to have food addiction. We also found that FR and EF, instead of SR and SE, are abnormal between patients with obesity and PWS and healthy controls with obesity. Besides, the eating behavior patterns, such as FR, EF, SR, and mYFAS-C 2.0 scores, exhibit abnormalities compared to healthy controls even before the onset of overweight or obesity. Through the refinement of the animal model, we found that *SNORD116^-/-^* mice also exhibited hedonic feeding in juveniles, which is consistent with the conclusion of this study (Data not shown). Collectively, these phenomena indicate that early changes in FR, EF, and food addiction may be correlated with hyperphagia and subsequent weight gain in patients with PWS. Taken together, the abnormality of FR may play a critical role in hyperphagia in patients with PWS.

Growth hormone (GH) treatment for PWS has been widely used since it was first approved in the United States in 2000 and in Europe in 2001. Growth hormone therapy has been shown to have positive effects on linear growth, body composition, and motor and cognitive development (41–43). Multiple studies have confirmed that early initiation of GH therapy is expected to be more effective than later treatment without increased adverse effects. (33, 44, 45) Here, GH therapy has been proven to be an independent factor influencing weight, and continuous GH therapy helps to maintain weight, with earlier treatment yielding more significant positive effects. This finding is consistent with previous studies showing that GH treatment not only helps improve patients’ height and muscle mass but also reduces fat (46).

We also found that food responsiveness (FR) contributes to patients’ body weight. The “food responsiveness” in the CEBQ refers to the level of interest and reaction an individual has toward food, also known as food cue reactivity. This responsiveness can manifest as a desire for food and a sensitivity to food-related environments. Children with high food responsiveness often exhibit an intense desire to eat upon exposure to visual or olfactory food cues, showing significant interest and cravings for food, regardless of their hunger state. These behaviors closely mirror clinical characteristics observed in patients with PWS. Abnormal FR means exaggerated responses to food cues and rewards. Extensive research has shown that brain regions central to reward seeking modulate feeding. For example, the nucleus accumbens (NAc) and ventral pallidum (VP) are two major interconnected reward-processing structures that contribute to food intake and obesity (47). The orbitofrontal cortex (OFC) acts as an integrative hub for orchestrating motivated feeding behavior in the prefrontal cortex (48). The following brain regions together constitute the dopamine system: paraventricular thalamus, laterodorsal tegmental area, amygdala, ventral tegmental area (VTA), and NAc, which mediate feeding motivation (49). Collectively, these brain regions and the dopamine system underpin food addiction and food cue reactivity. Moreover, fMRI in individuals with PWS reveals greater activation of food pictures in the orbitofrontal cortex, prefrontal cortex, amygdala, and hippocampus, which is partly consistent with food cue reactivity-related brain regions (38, 39, 50, 51). This consistency suggests high FR might be a key factor to hyperphagia in patients with PWS.

To sum up, High food responsiveness may be a target for the treatment of hyperphagia. Despite the significant effects of GH therapy on weight management, it does not improve patients’ eating behavior alterations. Recent studies increasingly suggest that GH plays a role in neural differentiation, neural protection, signal transduction, and other related processes. (34, 52, 53) The ineffectiveness of growth hormone in improving eating behavior in patients with PWS demonstrates that alterations of eating behavior in patients with PWS may not be primarily due to the GH deficiency effects on neuronal function, but rather primarily attributed to the effects of gene deletion. This result suggests that solely relying on GH therapy is insufficient to comprehensively manage the dietary behavior of patients with PWS.

Drugs that are currently in clinical trials and their outcomes for appetite control are detailed by Christoffersen (54). However, there are currently no clinical medications available for appetite control. Randomized controlled trials (RCTs) have shown that Emotional Freedom Techniques (EFT) and Cognitive Behavioural Therapy (CBT) exhibit comparable efficacy in reducing food cravings, decreasing “ responsiveness to food in the environment “, and improving dietary restraint. Following intervention, participants demonstrated reduced scores, which approximated the levels of non-clinical community samples (55). Additionally, meta-analyses of RCTs indicated that short-term and long-term Mindfulness-based Interventions (MBIs) exerted varying degrees of effect on weight improvement and binge eating disorder (BED) symptom reduction, respectively (56). Considering high food responsiveness in patients with PWS, psychological therapies, such as cognitive-behavioral therapy and mindfulness-based interventions, may effectively improve eating behaviors in patients with PWS. Moreover, studies have shown that early improvement in eating behaviors might have long-term benefits for weight management (57). To improve the physical and mental health of patients and reduce the burden on parents and caregivers, the involvement of behavioral interventions and psychotherapeutic interventions, as early as possible, may be helpful for patients to better manage hyperphagia-related behaviors. Future clinical trials could evaluate the efficacy of interventions by general practitioners or psychologists among patients with PWS, assessing both timing (early vs. late) and duration (short vs. long) of intervention on improving hyperphagia-related behaviors and reducing obesity.

The present study primarily focused on Chinese participants in China. Future research could be conducted in other countries and among diverse ethnic groups to further enhance the generalizability of the study findings. Besides, the assessment of eating behaviors in our study was primarily conducted via questionnaires. Although we incorporated follow-up calls to enhance the reliability of the information obtained, response bias (e.g., parental subjective perceptions or recall biases) may still exist. Other more objective research methods, such as fMRI, combined with questionnaires may be considered in the future. Studies investigating the environmental determinants of childhood obesity and appetite demonstrated that food responsiveness (FR), enjoyment of food (EF), satiety responsiveness (SE), and slowness in eating (SR) are not significantly correlated with early feeding practices, birth weight, parental educational levels, cooking oil consumption, or annual household income (26). Only maternal BMI was negatively correlated with SE and SR. However, socioeconomic factors, such as caregiver education and social environment, may still exert an influence on eating behaviors. Additional evidence is needed in future research to clarify these potential associations with eating behaviors in PWS patients. Due to the complexity and difficulty of obtaining accurate data on diet types and energy intake, we could not establish a linear relationship between eating behavior scores and energy intake in patients with PWS. Clinical studies that further clarify the relationship between eating behavior scores and energy intake in patients with PWS are essential in the future.

## 5 Conclusions

In summary, this study indicates that GH treatment and food responsiveness are significant factors influencing hyperphagia and body weight in patients with PWS. GH treatment is crucial for early intervention and weight maintenance, but additional therapies are needed to address food responsiveness. Early involvement of psychotherapeutic interventions may help patients to better manage hyperphagia-related behaviors. Additionally, developing more effective intervention strategies to improve food responsiveness is a crucial direction for appetite control in patients with PWS in the future. Ultimately, a combined strategy—integrating biological interventions like GH treatment (for early weight management) and behavioral approaches (like psychotherapeutic support)—is essential for effectively and sustainably addressing hyperphagia and body weight in patients with PWS. Longitudinal studies are needed to explore the impact of psychological therapies on appetite in patients with PWS in the future.

## Data Availability

The original contributions presented in the study are included in the article/**Supplementary Material**. Further inquiries can be directed to the corresponding author.
