# Supplementary material for: Food responsiveness, addiction, and hyperphagia in Prader-Willi syndrome: a cross-sectional study of 210 Chinese patients
Source: Front Endocrinol (Lausanne). 2025 Oct 23;16:1665040. doi: 10.3389/fendo.2025.1665040 (PMC12588852; doi:10.3389/fendo.2025.1665040)
Supplement: Supplementary file 1 [file DataSheet1.docx]

**Supporting information**

**Association among food responsiveness, food addition and hyperphagia in Prader–Willi syndrome：a cross-sectional analysis of 210 patients from 26 provinces in China**

**Figure S1** Differences in the scores of the CEBQ subscales and the mYFAS-C 2.0 among age groups.

**Table S1** Pearson’s correlation between demographic characteristics, the CEBQ subscales and mYFAS-C 2.0

**Table S2** Differences of weight in CEBQ and mYFAS-C 2.0

**Table S3** Age differences in the CEBQ and mYFAS-C 2.0

**Table S4** Effects of gender, age, genetic subtype and GH therapy on weight


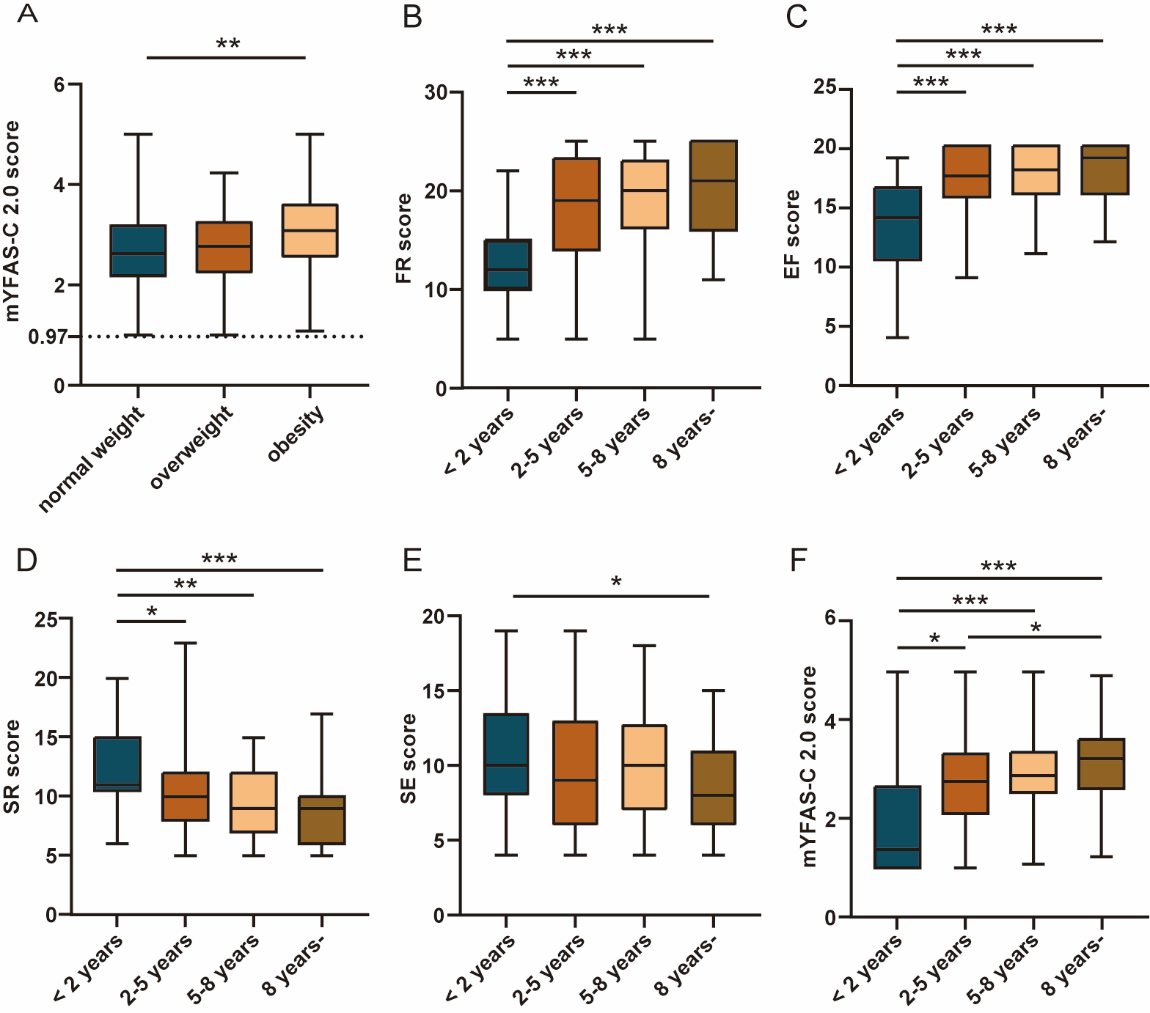


**Figure S1** | Differences in the scores of the CEBQ subscales and the mYFAS-C 2.0 among age groups. **P* < 0.05, ***P* < 0.01, ****P* < 0.001.

**Table S1** | Pearson’s correlation between demographic characteristics, the CEBQ subscales, and mYFAS-C 2.0

|  | **BMI-Z** | **Age(years)** | **GH therapy** | **mYFAS** | **FR** | **EF** | | **SR** | | **SE** | | **EOE** | | **FF** |  |
| --- | --- | --- | --- | --- | --- | --- | --- | --- | --- | --- | --- | --- | --- | --- | --- |
| **BMI-Z** | - |  |  |  |  | |  | |  | |  | |  | |  |
| **Age(years)** | 0.241** | - |  |  |  | |  | |  | |  | |  | |  |
| **GH therapy** | -0.304** | -0.492** | - |  |  | |  | |  | |  | |  | |  |
| **mYFAS** | 0.296** | 0.263** | -0.207** | - |  | |  | |  | |  | |  | |  |
| **FR** | 0.311** | -0.215** | -0.132 | 0.705** | - | |  | |  | |  | |  | |  |
| **EF** | 0.231** | 0.179** | -0.034 | 0.624** | 0.767** | | - | |  | |  | |  | |  |
| **SR** | -0.158** | -0.169** | 0.04 | -0.367** | -0.577** | | -0.609** | | - | |  | |  | |  |
| **SE** | -0.214** | -0.095 | -0.008 | -0.261** | -0.302** | | -0.317** | | 0.42** | | - | |  | |  |
| **EOE** | 0.235** | 0.196** | -0.124 | 0.612** | 0.622** | | 0.489** | | -0.325** | | -0.236** | | - | |  |
| **FF** | -0.153** | -0.232** | 0.034 | -0.281** | -0.535** | | -0.466** | | 0.538** | | 0.339** | | -0.297** | | - |

Note: **P* < 0.05. ***P* < 0.01.

FR: food responsiveness, EF: enjoyment of food, SR: satiety responsiveness, SE: slowness in eating, EOE: emotional over-eating, FF: food fussiness.

|  | **Normal weight** | **Overweight** | **Obesity** | **H（F）** | **P** |
| --- | --- | --- | --- | --- | --- |
| **CEBQ(median,25%~75%)** |  |  |  |  |  |
| FR | 17(13.75~22.25) | 19(16.5~22.5) | 20.5(17~24.25) | 8.988 | 0.011* |
| EF | 17.5(14.75~20) | 18(16~20) | 19(16~20) | 4.94 | 0.085 |
| SR | 9.5(7~12.25) | 9(6.5~10.5) | 9(7~11) | 2.391 | 0.302 |
| SE | 10(7.75~13) | 10(6.5~10) | 8(6~11) | 7.728 | 0.021* |
| EOE | 10.5(8~13) | 11(10~12) | 12(10~15) | 10.724 | 0.005** |
| EUE（mean,SD） | 9.83(2.91) | 10(2.89) | 10.34(2.53) | 0.676 | 0.51# |
| DD | 7(5~8) | 7(5~8) | 7(6~8) | 2.119 | 0.347 |
| FF（mean,SD） | 14.07(4.03) | 12.92(2.94) | 13.94(4.62) | 1.245 | 0.294## |
| **mYFAS-C 2.0(（mean,SD）** | 2.67(0.85) | 2.77(0.81) | 3.09(0.79) | 5.326 | 0.006#** |

**Table S2** | Differences of weight in CEBQ and mYFAS-C 2.0

Note: ^#^ One-way ANOVA. ^##^ Welch test. The rest are Kruskal-Wallis test. **P* < 0.05. ***P* < 0.01

**Table S3** | Age differences in the CEBQ and mYFAS-C 2.0 (N=210)

|  | **<2years** | **2-5years** | **5-8years** | **8years-** | **H** | **P** |
| --- | --- | --- | --- | --- | --- | --- |
| **CEBQ(median,25%~75%)** |  |  |  |  |  |  |
| FR | 12(10~15) | 19(14~23.25) | 20(16.25~23) | 21(16~25) | 38.476 | <0.001*** |
| EF | 14(10.5~16.5) | 17.5(15.75~20) | 18(16~20) | 19(16~20) | 33.707 | <0.001*** |
| SR | 11(10.5~15) | 10(8~12) | 9(7~12) | 9(6~10) | 23.504 | <0.001*** |
| SE | 10(8~13.5) | 9(6~13) | 10(7~12.75) | 8(6~11) | 10.078 | 0.018* |
| EOE | 8(5.5~12) | 11(8~13) | 12(9~13) | 12(9~15) | 18.721 | <0.001*** |
| EUE | 10(8~11.5) | 11(8.75~12) | 10(8~11) | 10(8~12) | 4.801 | 0.187 |
| DD | 6(4~7) | 6(5~8) | 7(5~8) | 7(6~9) | 6.919 | 0.075 |
| FF | 17(14~18.5) | 15(11~18) | 14(12~16.75) | 12(10~15) | 20.432 | <0.001*** |
| **mYFAS-C 2.0(median,25%~75%)** | 1.38(1~2.65) | 2.77(2.12~3.33) | 2.88(2.54~3.37) | 3.23(2.62~3.62) | 30.199 | <0.001*** |

Note: The data are processed by Kruskal-Wallis test. **P* < 0.05. ***P* < 0.01, ****P* < 0.001

**Table S4** | Effects of gender, age, genetic subtype and GH therapy on body weight

|  | **Normal weight** | **Overweight** | **Obesity** | **H** | **P** |
| --- | --- | --- | --- | --- | --- |
| **Gender (N, %)** |  |  |  | 2.733 | P=0.098 |
| Male | 43(41%) | 14(13.3%) | 48(45.7%) |  |  |
| Female | 33(31.4%) | 12(11.4%) | 60(57.1%) |  |  |
| **Age group (N, %)** |  |  |  | 52.216 | P<0.001*** |
| < 2years | 22(88%) | 1(4%) | 2(8%) |  |  |
| 2 - 5years | 31(50%) | 10(16.1%) | 21(33.9%) |  |  |
| 5 - 8years | 12(21.4%) | 10(17.9%) | 34(60.7%) |  |  |
| 8years- | 11(16.4%) | 5(7.5%) | 51(76.1%) |  |  |
| **Genetic subtype (N, %)** |  |  |  | 6.027 | P = 0.014* |
| Deletion | 50(32.3%) | 20(12.9%) | 85(54.8%) |  |  |
| Non deletion | 18(54.5%) | 4(12.1%) | 11(33.3%) |  |  |
| Not clear | 8(36.4%) | 2(9.1%) | 12(54.5%) |  |  |
| **GH therapy (N, %)** |  |  |  | 21.467 | P < 0.001*** |
| never | 5(20%) | 1(4%) | 19(76%) |  |  |
| ever | 6(13.3%) | 7(15.6%) | 32(71.1%) |  |  |
| now | 65(46.4%) | 18(12.9%) | 57(51.4%) |  |  |

Note : The data are processed by Kruskal-Wallis test. **P* < 0.05. ***P* < 0.01, ****P* < 0.001
